# Willingness of community-recruited men who have sex with men in Washington, DC to use long-acting injectable HIV pre-exposure prophylaxis

**DOI:** 10.1371/journal.pone.0183521

**Published:** 2017-08-21

**Authors:** Matthew E. Levy, Rudy Patrick, Jonjelyn Gamble, Anthony Rawls, Jenevieve Opoku, Manya Magnus, Michael Kharfen, Alan E. Greenberg, Irene Kuo

**Affiliations:** 1 Department of Epidemiology and Biostatistics, Milken Institute School of Public Health at the George Washington University, Washington, DC, United States of America; 2 HIV/AIDS, Hepatitis, STD, and TB Administration, District of Columbia Department of Health, Washington, DC, United States of America; Thai Red Cross AIDS Research Center, THAILAND

## Abstract

**Objectives:**

Clinical trials are currently investigating the safety and efficacy of long-acting injectable (LAI) agents as HIV pre-exposure prophylaxis (PrEP). Using National HIV Behavioral Surveillance data, we assessed the self-reported willingness of men who have sex with men (MSM) to use LAI PrEP and their preference for LAI versus daily oral PrEP.

**Methods:**

In 2014, venue-based sampling was used to recruit MSM aged ≥18 years in Washington, DC. Participants completed an interviewer-administered survey followed by voluntary HIV testing. This analysis included MSM who self-reported negative/unknown HIV status at study entry. Correlates of being “very likely” to use LAI PrEP and preferring it to daily oral PrEP were identified using multivariable logistic regression.

**Results:**

Of 314 participants who self-reported negative/unknown HIV status, 50% were <30 years old, 41% were non-Hispanic Black, 37% were non-Hispanic White, and 14% were Hispanic. If LAI PrEP were offered for free or covered by health insurance, 62% were very likely, 25% were somewhat likely, and 12% were unlikely to use it. Regarding preferred PrEP modality, 67% chose LAI PrEP, 24% chose oral PrEP, and 9% chose neither. Correlates of being very likely versus somewhat likely/unlikely to use LAI PrEP included age <30 years (aOR 1.64; 95% CI 1.00–2.68), reporting ≥6 (vs. 1) sex partners in the last year (aOR 2.60; 95% CI 1.22–5.53), previous oral PrEP use (aOR 3.67; 95% CI 1.20–11.24), and being newly identified as HIV-infected during study testing (aOR 4.83; 95% CI 1.03–22.67). Black (vs. White) men (aOR 0.48; 95% CI 0.24–0.96) and men with an income of <$20,000 (vs. ≥$75,000; aOR 0.37; 95% CI 0.15–0.93) were less likely to prefer LAI to oral PrEP.

**Conclusions:**

If LAI PrEP were found to be efficacious, its addition to the HIV prevention toolkit could facilitate more complete PrEP coverage among MSM at risk for HIV.

## Introduction

In the United States (US), the HIV epidemic continues to disproportionately affect men who have sex with men (MSM), particularly younger men and Black and Latino/Hispanic men [1]. In 2012, the US Federal Drug Administration (FDA) approved daily oral pre-exposure prophylaxis (PrEP) with emtricitabine/tenofovir disoproxil fumarate (FTC/TDF) for HIV prevention based on results of the iPrex study, which found that PrEP reduced HIV incidence by 44% among MSM [2]. Daily oral PrEP was up to 92% effective, however, among participants with detectable drug in their blood, demonstrating a dramatically higher effectiveness for men who were adherent to the once-daily pill regimen [2]. The Centers for Disease Control and Prevention (CDC) have since defined indications for PrEP use by HIV-uninfected adult MSM who are not in a monogamous relationship with a recently tested HIV-uninfected partner, recommending PrEP for MSM in an ongoing sexual relationship with an HIV-infected male partner or for MSM who have had any anal sex without a condom in the last six months or any sexually transmitted infection (STI) diagnosed or reported in the last six months [3]. Low adherence has prevented participants in various studies from achieving protection from HIV infection using PrEP, as its effectiveness is limited by one’s willingness and ability to take a daily pill [4]. Previous research has found that MSM expressed concerns about the necessity of taking a pill every day and about the potential for other people including sexual partners to discover their PrEP use [5–7]. In the iPrex study, barriers to PrEP adherence among MSM included changes in routine, side effects/intercurrent illnesses, and stress [8]. Daily oral drug regimens can be challenging in general due to pill fatigue and the ongoing need to remember to take medication daily [7], particularly among younger populations, as PrEP adherence was found to be low in one PrEP trial of young MSM [9].

Clinical trials are underway to investigate the safety and efficacy of long-acting injectable (LAI) agents as PrEP, which could overcome adherence challenges associated with daily oral PrEP and maintain adequate levels of PrEP in the blood for a longer period of time following drug administration [10, 11]. Three Phase 2 clinical trials to evaluate the safety and tolerability of LAI PrEP are either ongoing or completed (NCT02076178, NCT02178800, NCT02165202), and a Phase 2b/3 trial to evaluate the efficacy of LAI PrEP began enrolling participants in December 2016 (NCT02720094). In that study (HPTN 083), a long-acting formulation of the integrase inhibitor cabotegravir is administered via intramuscular injection at two time points one month apart followed by every two months thereafter [12]. If LAI PrEP is shown to be efficacious, it has the potential to increase PrEP coverage among MSM–if men are willing to use it. In the Phase 2 ECLAIR study, 79% of men at low risk of HIV infection who received LAI cabotegravir were willing to continue with the study product after receiving three injections in three-month dosing intervals [13]. To date, several other studies have found moderate to high willingness to use LAI PrEP based on hypothetical questions asked of MSM: 53% of a national Internet-based sample of MSM were willing to use LAI PrEP every one to three months [14], 43% and 54% of another national study sample of MSM were willing to use LAI PrEP every one or three month(s), respectively [15], and 81% of a sample of young MSM in New York City were either probably or definitely willing to use LAI PrEP every three months [16]. Several studies have also found that MSM preferred LAI PrEP to daily oral PrEP [15, 16], although one study found a more consistent preference for daily oral PrEP [17]. However, that study provided participants with three other HIV prevention options in addition to daily oral PrEP and LAI PrEP–condoms and two subcutaneous implant options–and considered participants to have preferred one of the five options if it was consistently selected over all other options in pairwise comparisons; thus, a direct comparison between preference for daily oral versus LAI PrEP was not possible, as many participants consistently preferred condoms or implants [17].

While these findings are promising, initial studies of willingness to use LAI PrEP have been conducted among study samples with limited representativeness of MSM at greatest risk of HIV who are not currently receiving or adhering to existing HIV prevention interventions, and thus who stand to benefit the most from the potential availability of new PrEP modalities such as LAI PrEP. Populations of Black MSM continue to experience disproportionately high HIV incidence rates relative to those among MSM of other races/ethnicities, yet only 5% and 7% of men in two of these studies were Black [14, 15], and 22% in a third study were non-White (proportions for specific races/ethnicities were not provided) [17]. One other study was conducted among a more diverse sample of young MSM, though it comprised of men who had already been engaged in HIV prevention services as research participants in a longitudinal cohort study for several years [16]. In order to supplement these findings, we assessed willingness to use LAI PrEP and preference for LAI PrEP versus daily oral PrEP among a diverse community-recruited sample of MSM in a high-prevalence city, so as to maximize the generalizability of findings regarding LAI PrEP to key target populations. This research offers insight into the level of interest in LAI PrEP among MSM and can inform strategies for scaling up LAI PrEP to target populations if it is found to be safe and efficacious.

## Methods

### Study design and sampling

Data were used from the fourth data collection cycle conducted among MSM in Washington, DC for the Centers for Disease Control and Prevention (CDC) National HIV Behavioral Surveillance (NHBS) study [18]. Recruitment was completed between August and December 2014 using venue-based time-space sampling. Recruitment methods for NHBS MSM cycles have been described in detail elsewhere [19]. In brief, a sampling frame of eligible venues was generated that included all potential public venues from which MSM could be recruited. Eligible venues were defined as those at which at least 50% of male attendees were MSM, and were identified based on formative research, including key informant and street intercept interviews. On a monthly basis, a calendar of recruitment events was created by randomly selecting venues and corresponding days and times of the week. At each recruitment event, men attending the venue were systematically approached for recruitment and screening for the study.

Eligibility criteria included being at least 18 years old, being born male and identifying as male, reporting oral or anal sex with a male partner in the last 12 months, being able to complete the survey in English, and living in the Washington, DC Metropolitan Statistical Area. Once study staff determined that a potential participant was eligible, individuals provided informed consent and completed an interviewer-administrated behavioral survey. All participants were offered rapid HIV testing (Clearview Complete HIV 1/2, Alere, Waltham, MA), and preliminary HIV-positive results were confirmed via Western Blot. Test results from the HIV rapid test were returned after completion of the behavioral survey; thus, we defined being newly identified as HIV-infected during study testing (i.e., having tested positive without having reported prior knowledge of his HIV infection) as a variable of interest. All individuals screening preliminary positive were pre-emptively referred to HIV care. Study participation was anonymous, and participants were offered incentives of $25 for completing the survey and $10 for HIV testing.

All study participants provided informed verbal consent for the behavioral survey and the HIV test, which was documented on paper by the interviewers on a copy of the consent form that was retained by study staff and electronically in the study tablet. Verbal instead of written consent was obtained due to the sensitive nature of questions and also to maintain participant anonymity. The NHBS study protocol was reviewed and approved by the District of Columbia Department of Health and George Washington University Institutional Review Boards.

### Measures

The NHBS core survey assessed demographics, sexual and drug use behaviors, health seeking behaviors, and PrEP use. In addition, we included local questions on willingness to use LAI PrEP and preference for LAI PrEP versus daily oral PrEP. To measure willingness to use LAI PrEP, participants were asked, “If an anti-HIV injection or shot that you had to take every 1 to 3 months existed that would prevent you from getting HIV was available for free or was covered by your health insurance, how likely would you be to take it?” with the following response options: “very likely,” “somewhat likely,” or “not at all likely.” To assess one’s preference between LAI PrEP and daily oral PrEP, participants were asked, “If you had the choice between taking a once-daily anti-HIV oral pill or an anti-HIV injection once every 3 months, which one would you prefer, assuming the cost was the same?” Response options included “daily oral pill,” “every three month injection,” or “neither.”

### Data analysis

Analyses were restricted to participants who self-reported HIV-negative or unknown HIV status prior to HIV testing as part of the study. For descriptive purposes, frequencies and percentages of response options were reported for each variable, among all participants and stratified by one’s willingness to use LAI PrEP and by one’s preference for LAI versus daily oral PrEP. For analytic purposes, we dichotomized the outcome of willingness to use LAI PrEP as being very likely versus somewhat or not at all likely to use LAI PrEP, which is consistent with the categorization scheme for willingness to use PrEP that we have utilized previously [20]. We used chi-square or Fisher’s exact tests as well as logistic regression models to generate unadjusted and adjusted odds ratios and 95% confidence intervals for associations between covariates and each of two outcome variables: being very likely to use LAI PrEP and, among participants who had a preference, preferring LAI to daily oral PrEP. Variables with p<0.20 in univariable models were considered for inclusion in multivariable models and were subsequently eliminated using a manual stepwise method. Given the moderate sample size, we retained all covariates with p<0.10 to ensure adjustment for potential confounding variables, although statistical significance was defined as p<0.05. We also retained race/ethnicity in multivariable models regardless of whether p<0.10 since it was a key demographic variable of interest. All statistical analyses were conducted using SAS 9.4 (SAS Institute Inc., Cary, NC).

## Results

Of 314 MSM who self-reported HIV-negative or unknown status, 50% were between 18–29 years old (median 29.5 years; IQR 25–35; range 18–66), 41% were non-Hispanic Black, 37% were non-Hispanic White, and 14% were Latino/Hispanic (Table 1). Forty percent of participants reported six or more male sex partners in the last 12 months and 43% reported condomless anal sex at their most recent sex encounter. In terms of health care access and health seeking behaviors, 91% of participants reported having health insurance, 83% reported seeing a doctor in the last 12 months, 77% reported testing for HIV in the last 12 months, and 9% reported ever having used oral PrEP. Based on HIV testing conducted as part of the study visit, 5% of men were newly identified as HIV-infected. One participant who did not know his willingness or preference was excluded from subsequent analyses.

**Table 1:** Descriptive characteristics and correlates of being very likely to use long-acting injectable PrEP among men who have sex with men recruited for NHBS in Washington, DC who self-reported HIV-negative or unknown status, 2014 (n = 314).

	All participants	Stratified by Willingness to Use LAI PrEP			Logistic Regression Modeling	
	(n = 314)	Very likely (n = 196)	Somewhat/not likely (n = 118) ^a^		Being very likely vs. somewhat/not likely to use LAI PrEP	
	n (%)	n (%)	n (%)	χ^2^, p-value	OR (95% CI)	aOR (95% CI)
**Age <30 years**	157 (50.0)	106 (54.1)	51 (43.2)	3.5, 0.062	1.55 (0.98–2.45)	1.64 (1.00–2.68)*
**Race/ethnicity**				2.2, 0.34		
**Non-Hispanic Black**	114 (40.7)	77 (39.3)	38 (32.2)		1.26 (0.74–2.13)	1.32 (0.74–2.37)
**Other ^b^**	27 (8.6)	40 (20.4)	31 (26.3)		0.80 (0.44–1.44)	0.77 (0.41–1.44)
**Non-Hispanic White**	128 (36.6)	79 (40.3)	49 (41.5)		1.00 (—)	1.00 (—)
**Annual household income ^c^**				0.5, 0.93		
**<$20,000**	34 (10.8)	21 (10.8)	13 (11.0)		0.98 (0.45–2.12)	†
**$20,000 - $39,999**	42 (13.4)	28 (14.4)	14 (11.9)		1.21 (0.58–2.51)	†
**$40,000 - $74,999**	99 (31.5)	60 (30.8)	39 (33.0)		0.93 (0.55–1.58)	†
**≥$75,000**	138 (43.9)	86 (44.1)	52 (44.1)		1.00 (—)	†
**Non-injection recreational drug use, last 12 months**	165 (52.5)	107 (54.6)	58 (49.2)	0.9, 0.35	1.24 (0.79–1.97)	†
**Number of male sex partners, last 12 months**				10.2, **0.006**		
**1**	42 (13.4)	20 (10.2)	22 (18.6)		1.00 (—)	1.00 (—)
**2–5**	146 (46.5)	85 (43.4)	61 (51.7)		1.53 (0.77–3.05)	1.26 (0.62–2.58)
**≥6**	126 (40.1)	91 (46.4)	35 (29.7)		2.86 (1.39–5.88)**	2.60 (1.22–5.53)*
**Insertive anal sex, last sex encounter**	157 (50.0)	100 (51.0)	57 (48.3)	0.2, 0.64	1.12 (0.71–1.76)	†
**Condomless anal, last anal sex encounter**	133 (42.6)	77 (39.5)	56 (47.9)	2.1, 0.15	0.71 (0.45–1.13)	†
**Had casual sex partner(s), last 12 months**	267 (85.0)	172 (87.8)	95 (80.5)	3.0, 0.081	1.74 (0.93–3.24)	†
**Received an HIV test, last 12 months**	243 (77.4)	154 (78.6)	89 (75.4)	0.4, 0.52	1.20 (0.70–2.05)	†
**Has health insurance**	285 (90.8)	177 (90.3)	108 (91.5)	0.1, 0.72	0.86 (0.39–1.92)	†
**Has seen a doctor, last 12 months**	260 (82.8)	166 (84.7)	94 (79.7)	1.3, 0.25	1.41 (0.78–2.56)	†
**Ever used oral PrEP**	28 (8.9)	24 (12.2)	4 (3.4)	**0.007** ^d^	3.98 (1.34–11.76)*	3.67 (1.20–11.24)*
**Newly identified as HIV-infected during NHBS study visit**	15 (4.8)	13 (6.6)	2 (1.7)	0.056 ^d^	4.12 (0.91–18.58)	4.83 (1.03–22.67)*

NHBS, National HIV Behavioral Surveillance; PrEP, pre-exposure prophylaxis; LAI, long-acting injectable; OR, odds ratio; CI, confidence interval; aOR, adjusted odds ratio.

*p<0.05

**p<0.01.

† Not included in the multivariable model because p>0.10 in manual stepwise regression modeling.

^a^ One participant who reported unknown willingness to use LAI PrEP was categorized as being somewhat/not likely to use LAI PrEP for purposes of this analysis.

^b^ Participants with race/ethnicity classified as “other” included 19 multiracial participants, four Asian participants, three Native Hawaiian participants, and one American Indian participant.

^c^ A response for annual household income was missing for one participant.

^d^ These p-values were obtained using Fisher’s exact test.

**Fig 1:**
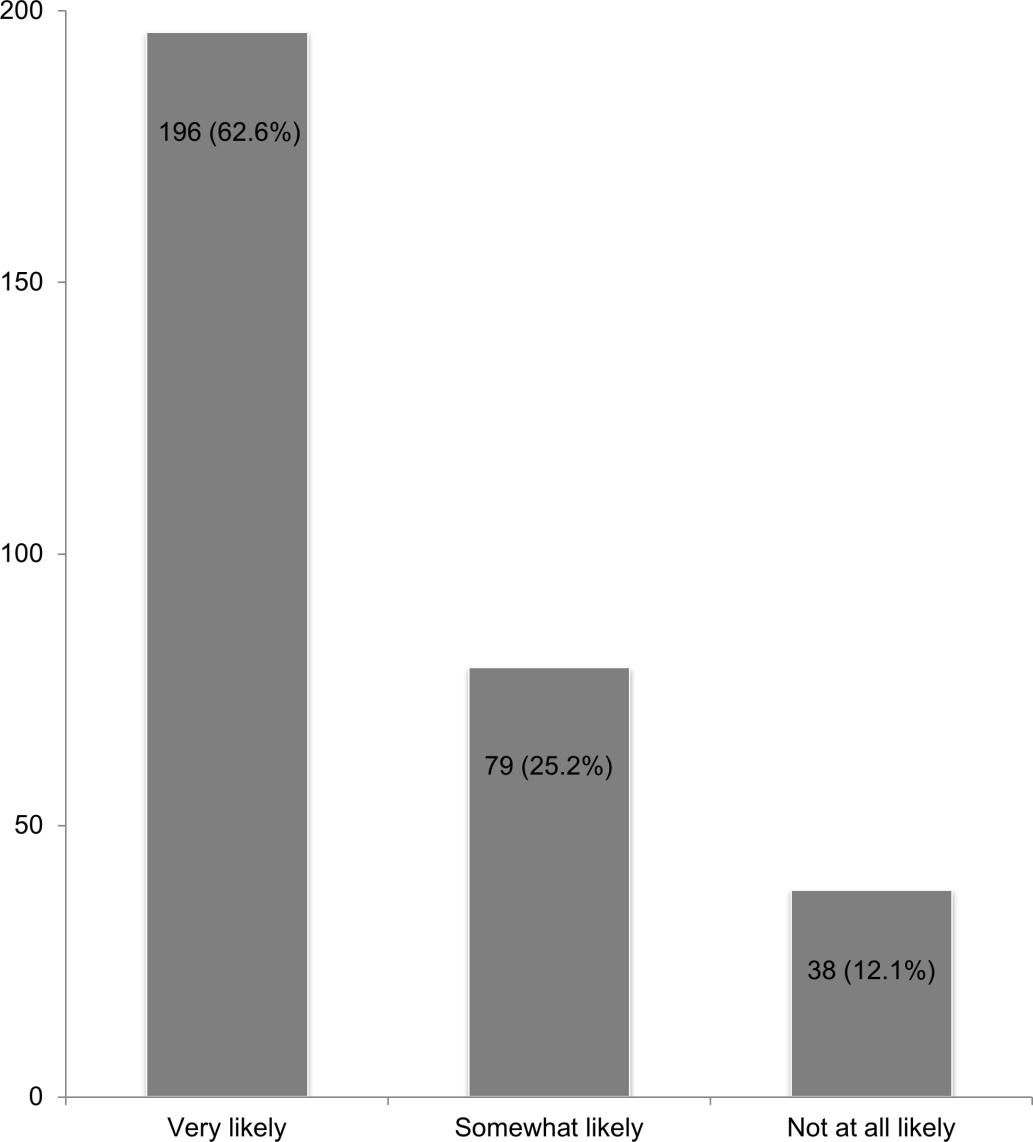
Willingness to use long-acting injectable PrEP among men who have sex with men (n = 313). One participant who did not know his willingness was excluded.

Regarding willingness to use LAI PrEP if available for free or covered by health insurance, a majority of participants were willing to use LAI PrEP– 63% of participants were very likely to use it, while 25% were somewhat likely and 12% were not at all likely to use it (Fig 1). Among racial/ethnic groups, 68% of non-Hispanic Black men, 62% of non-Hispanic White men, and 59% of Latino/Hispanic men were very willing to use LAI PrEP (χ^2^ = 1.1; p = 0.57). When stratified by age group, 69% of men aged 18–24, 67% of men aged 25–29, 56% of men aged 30–39, and 60% of men aged ≥40 were very likely to use it (χ^2^ = 4.0; p = 0.26).

**Fig 2:**
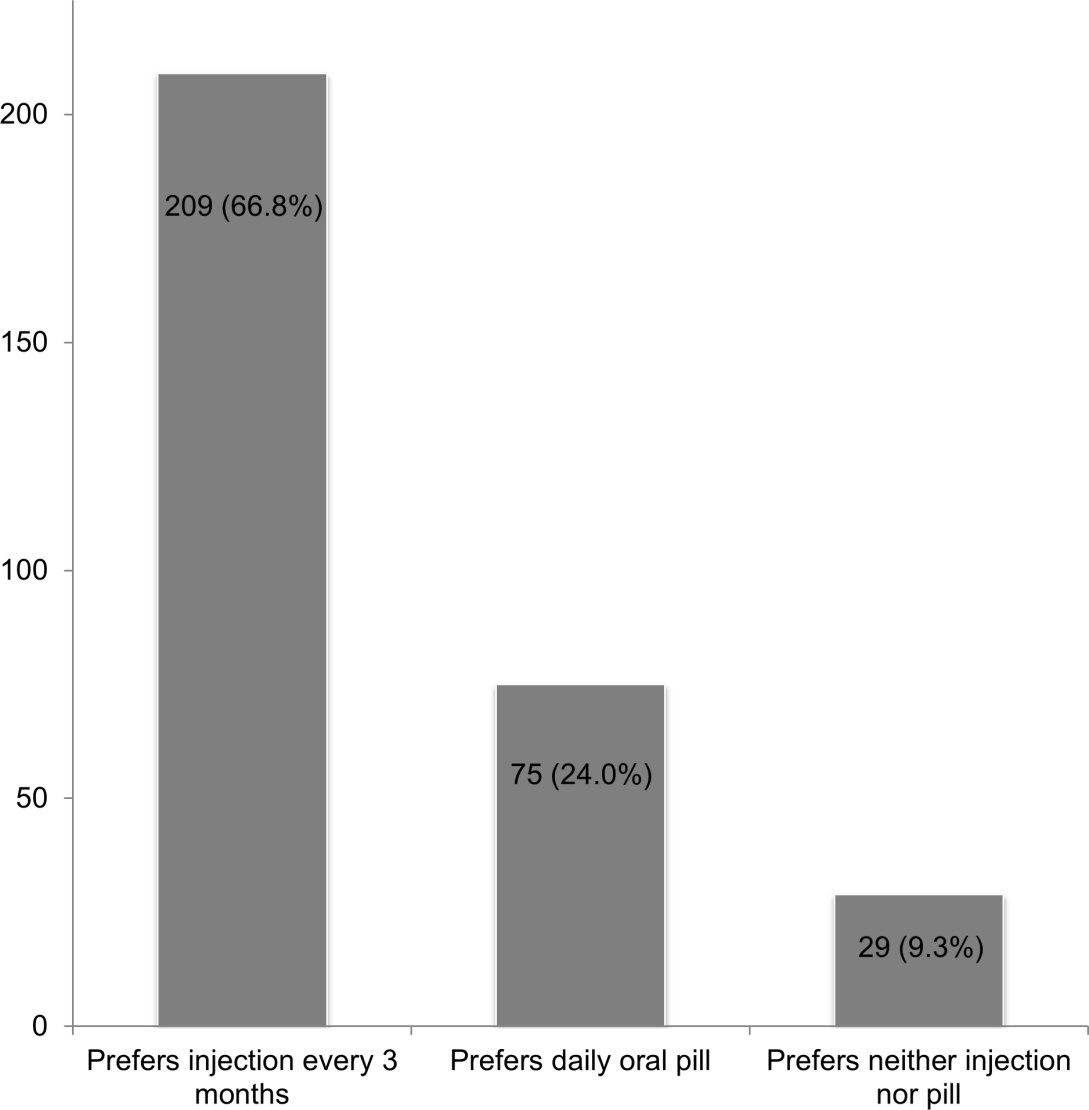
Preference for long-acting injectable versus daily oral PrEP among men who have sex with men (n = 313). One participant who did not know his preference was excluded.

Regarding one’s preference for LAI PrEP versus daily oral PrEP if the cost was equal, 67% preferred LAI PrEP, while 24% preferred daily oral PrEP and 9% preferred neither (Fig 2). Among racial/ethnic groups, 57% of non-Hispanic Black men, 76% of non-Hispanic White men, and 67% of Latino/Hispanic men preferred LAI to daily oral PrEP (χ^2^ = 10.1; p = 0.006). When stratified by age group, there was no difference in the preference of LAI PrEP by age: 66% of men aged 18–24, 67% of men aged 25–29, 67% of men aged 30–39, and 67% of men aged ≥40 preferred LAI PrEP (χ^2^ = 0.06; p>0.99).

In multivariable regression modeling, independent correlates of being very likely to use LAI PrEP included being <30 years old (aOR 1.64; 95% CI 1.00–2.68), having six or more (vs. one) sex partners in the last 12 months (aOR 2.60; 95% CI 1.22–5.53), having ever used oral PrEP (aOR 3.67; 95% CI 1.20–11.24), and being newly identified as HIV-infected based on HIV testing for the study (aOR 4.83; 95% CI 1.03–22.67) (Table 2). Regarding preference for type of PrEP, non-Hispanic Black men (aOR 0.48; 95% CI 0.24–0.96) and men with an annual household income <$20,000 (vs. ≥$75,000; aOR 0.37; 95% CI 0.15–0.93) were less likely to prefer LAI PrEP to daily oral PrEP. In *post-hoc* analyses, there were no significant differences by race/ethnicity in the possession of health insurance or in health-seeking behaviors including whether participants received an HIV test in the last year, saw a doctor in the last year, and ever used oral PrEP. Of the 15 participants who were newly identified as HIV-infected at their study visit, 13 participants reported being very willing to take LAI PrEP, and six of those participants preferred LAI PrEP to daily oral PrEP.

**Table 2:** Correlates of preferring long-acting injectable PrEP to daily oral PrEP among men who have sex with men who had a preference for PrEP modality (n = 284).

	Stratified by Preference for PrEP Modality			Logistic Regression Modeling	
	Prefers LAI PrEP (n = 209)	Prefers daily oral PrEP (n = 75)		Prefers LAI PrEP to daily oral PrEP	
	n (%)	n (%)	χ^2^, p-value	OR (95% CI)	aOR (95% CI)
**Age <30 years**	39 (52.0)	107 (49.8)	0.1, 0.74	0.91 (0.54–1.55)	†
**Race/ethnicity**			10.4, **0.006**		
**Non-Hispanic Black**	37 (49.3)	65 (31.1)		0.36 (0.19–0.68)**	0.48 (0.24–0.96)*
**Other**	18 (24.0)	47 (22.5)		0.54 (0.26–1.11)	0.61 (0.29–1.28)
**Non-Hispanic White**	20 (26.7)	97 (46.4)		1.00 (—)	1.00 (—)
**Annual household income**			6.5, 0.089		
**<$20,000**	12 (16.2)	15 (7.2)		0.34 (0.14–0.82)*	0.37 (0.15–0.93)*
**$20,000 - $39,999**	12 (16.2)	27 (12.9)		0.62 (0.28–1.38)	0.81 (0.34–1.89)
**$40,000 - $74,999**	23 (31.1)	69 (33.0)		0.83 (0.43–1.56)	0.92 (0.46–1.82)
**≥$75,000**	27 (36.5)	98 (46.9)		1.00 (—)	1.00 (—)
**Non-injection recreational drug use, last 12 months**	35 (46.7)	117 (56.0)	1.9, 0.17	1.45 (0.86–2.47)	†
**Number of male sex partners, last 12 months**			2.9, 0.24		
**1**	13 (17.3)	23 (11.0)		1.00 (—)	†
**2–5**	36 (48.0)	95 (45.5)		1.49 (0.68–3.26)	†
**≥6**	26 (34.7)	91 (43.5)		1.98 (0.88–4.44)	†
**Insertive anal sex, last sex encounter**	34 (45.3)	108 (51.7)	0.9, 0.35	1.29 (0.76–2.19)	†
**Condomless anal, last anal sex encounter**	25 (33.3)	94 (45.4)	3.3, 0.069	1.66 (0.96–2.89)	1.73 (0.96–3.13)
**Had casual sex partner(s), last 12 months**	58 (77.3)	183 (87.6)	4.5, **0.034**	2.06 (1.05–4.07)*	†
**Received an HIV test, last 12 months**	55 (73.3)	168 (80.4)	1.6, 0.20	1.49 (0.81–2.78)	†
**Has health insurance**	66 (88.0)	194 (92.8)	1.7, 0.20	1.76 (0.74–4.22)	†
**Has seen a doctor, last 12 months**	62 (82.7)	177 (84.7)	0.2, 0.68	1.16 (0.57–2.35)	†
**Ever used oral PrEP**	5 (6.7)	23 (11.0)	0.37 ^a^	1.73 (0.63–4.73)	†
**Newly identified as HIV-infected during NHBS study visit**	8 (10.7)	6 (2.9)	**0.012** ^a^	0.25 (0.08–0.74)*	0.34 (0.10–1.09)

PrEP, pre-exposure prophylaxis; LAI, long-acting injectable; OR, odds ratio; CI, confidence interval; aOR, adjusted odds ratio; NHBS, National HIV Behavioral Surveillance.

*p<0.05

**p<0.01.

† Not included in the multivariable model because p>0.10 in manual stepwise regression modeling.

^a^ These p-values were obtained using Fisher’s exact test.

## Discussion

Among a diverse community-recruited sample of MSM from a high-prevalence setting, a substantial percentage of participants reported that they would be willing to use LAI PrEP to prevent HIV infection if it were offered for free or covered by their health insurance, with 63% being very likely to use it and 88% being either very likely or somewhat likely to use it. Because HIV test results were returned after completion of the behavioral survey (after participants reported on willingness to use LAI PrEP), we were also able to specifically examine willingness among those who tested positive for HIV without having had previous knowledge of their serostatus. In fact, 13 of the 15 participants who were newly identified as HIV-infected reported that they would be very willing to use LAI PrEP if it were available, suggesting LAI PrEP might be a desirable prevention strategy for those at highest risk for HIV. Moreover, two-thirds of the total sample preferred using LAI PrEP to daily oral PrEP (or neither), which suggests that if LAI PrEP is found to be efficacious, its potential availability as a new HIV prevention intervention may facilitate more complete PrEP coverage among MSM at high risk of HIV infection.

In this sample, which was recruited using venue-based sampling and included a majority of men of color, high willingness to use LAI PrEP was more common than in studies of national samples of predominantly White MSM recruited using Internet-based methods: two Internet-based studies found that approximately half of participants were willing to use LAI PrEP in three-month dosing intervals, based on dichotomized measures of 5-point Likert scales [14, 15]. Another study of an urban sample of young MSM enrolled in a longitudinal cohort study found that a larger majority of participants (81%) were willing to use LAI PrEP, which was also based on a dichotomized measure of a 5-point Likert scale [16]. However, we found that willingness to use LAI PrEP was considerable among all racial/ethnic subgroups, and there was not a statistically significant difference in willingness by race/ethnicity. Although comparisons of proportions of participants who reported hypothetical willingness to use LAI PrEP across studies are inexact due to different survey measures and different study populations, it is promising that a majority of MSM in each of these studies reported being willing to use LAI PrEP.

In multivariable analysis, correlates of greater HIV risk were significantly associated with being very willing to use LAI PrEP. Specifically, being less than 30 years old, having a greater number of sexual partners in the last 12 months, and being newly identified as HIV-infected based on study testing were each independently associated with being very willing to use LAI PrEP, after adjusting for previous oral PrEP use. A previous study similarly found that young MSM with a greater number of recent sexual partners and a previous diagnosis of a sexually transmitted infection were more likely to be willing to use LAI PrEP [16]. On the whole, these findings provide evidence that LAI PrEP may be a particularly acceptable new PrEP modality among MSM at high risk of HIV infection.

In previous studies of MSM, findings regarding preference for LAI versus daily oral PrEP were mixed. When provided with the choice between the two PrEP modalities (or neither), the majority of participants in our sample and in one other study of MSM preferred LAI PrEP [16]. When men in another study were provided with these same response options plus two additional options–no preference or whichever is most effective–more participants expressed a preference for LAI PrEP than daily oral PrEP or whichever was most effective [15]. However, another study found that men tended to rank daily oral PrEP higher than LAI PrEP when asked to rank their preferred HIV prevention options from a list of current or hypothetical PrEP modalities, including daily oral PrEP, on demand oral PrEP, LAI PrEP, and penile and rectal gels [14]. Despite inconsistencies across studies in overall percentages of participants who preferred various PrEP modalities, these results taken together point to a large proportion of MSM who would prefer to receive periodic injections of long-acting agents as PrEP rather than take a daily oral pill.

Interestingly, although more than two-thirds of non-Hispanic Black men in this study reported being very willing to use LAI PrEP, non-Hispanic Black MSM were less likely to prefer LAI PrEP when asked to choose between LAI PrEP and daily oral PrEP. This paradoxical finding might be explained by a greater mistrust of medical care in Black communities and a potentially more medicalized perception of injections relative to pills [21, 22]. Reasons for differential preference in PrEP modality by race/ethnicity and household income when men were provided with a choice between LAI PrEP and oral PrEP MSM should be further explored. Similarly, despite the fact that participants who were newly identified as HIV-infected were more likely to report being very willing to use LAI PrEP compared with participants who had non-reactive HIV test results, those individuals were also paradoxically less likely to choose LAI PrEP to daily oral PrEP when asked about their preferred option, although this association was not significant in multivariable analysis. This finding warrants further evaluation and may in part be a consequence of the small absolute number of participants who were newly identified as HIV-infected. A lack of familiarity with LAI PrEP relative to daily oral PrEP may have also played a role, as men who were generally very willing to use PrEP regardless of the modality may have chosen the most familiar PrEP option when asked to report their preference.

Despite high interest in LAI PrEP, potential barriers to accessing LAI PrEP may be similar to those that have limited uptake of oral FTC/TDF among MSM, even if the need to adhere to a daily pill is eliminated. If LAI PrEP is eventually approved as an HIV prevention intervention, PrEP candidates would still need to access health care, discuss PrEP with a health care provider, receive a prescription, and remain adherent to periodic clinic-based visits, which is analogous to the care continuum for receiving daily oral PrEP [23]. Known barriers to daily oral PrEP use among MSM and other populations include lack of knowledge of how to access PrEP, out-of-pocket costs, discomfort with disclosing sexual behavior to health care providers, concerns regarding short- and long-term side effects, low perceived personal susceptibility to contracting HIV, concerns about partial effectiveness of PrEP, and fear of risk compensation such as decreased use of condoms after initiating PrEP [5, 24–27]. If LAI PrEP is shown to be efficacious, strategies that have been developed to increase access to, and uptake of, oral PrEP and other HIV prevention interventions can be adopted to facilitate uptake of LAI PrEP among MSM [28].

A limitation of this study is that LAI PrEP is an investigational research product that was presented to participants as a hypothetical form of PrEP. Willingness to use LAI PrEP may not predict actual future behavior, as decisions to initiate biomedical HIV prevention interventions are multifactorial. Although candidates for LAI PrEP would not have to adhere to a daily pill, they would have to adhere to regular clinic-based visits for periodic safety assessments and HIV testing. For assessing one’s preference between daily oral and LAI PrEP, LAI PrEP was described as an injection occurring once every three months. However, its dosing schedule is not yet finalized and LAI PrEP is currently being investigated as an injection administered every two months. The extent to which LAI PrEP is preferred may differ depending on the frequency of injections. It is important to acknowledge that we were unable to communicate the potential side effects of LAI PrEP or its actual efficacy, as results from clinical trials of LAI PrEP were not yet available at the time of data collection. Future studies investigating the acceptability of LAI PrEP for HIV prevention among target populations should provide up-to-date information based on results obtained from trials of LAI PrEP. Furthermore, participants in this study were recruited using venue-based sampling in a single city, so findings may not be generalizable to the general US population of MSM. Also, this study sample could generally access health care and demonstrated a high level of health-seeking behaviors. Since survey responses were obtained during interviewer-administered questionnaires, participants’ responses regarding interest in PrEP, sexual behaviors, and previous knowledge of HIV serostatus may have been influenced by social desirability bias. Despite these limitations, our assessment of willingness to use LAI PrEP among MSM in a high-prevalence setting can be useful for informing clinical development and potential future strategies to facilitate actual uptake.

With clinical trials underway to investigate the safety and efficacy of LAI antiretroviral agents as PrEP for HIV prevention, these results hold promise for the potential impact that LAI PrEP could have on reducing HIV incidence among MSM populations, particularly for men who do not find daily oral PrEP to be an acceptable HIV prevention method or who are unable to remain adherent to a daily pill regimen. If LAI PrEP is proven to be efficacious in the clinical trial setting, its effectiveness in communities will depend on successful scale-up approaches that minimize costs and facilitate access for those individuals at greatest risk of infection. Future studies, including those utilizing qualitative research methods, should investigate more nuanced perceptions of LAI PrEP among MSM, including perceived potential barriers to uptake, as studies to date have generally used several closed-ended questions to measure interest in LAI PrEP. In addition, research should be designed to gain a better understanding of the reasons for differences by racial subgroup in one’s reported preferred PrEP modality. It will also be important for studies to assess the extent to which individuals already using oral PrEP would elect to switch to LAI PrEP if it were also available. Our findings provide reassuring evidence that the current biomedical HIV prevention agenda regarding LAI PrEP is focused on a novel prevention intervention that is likely to be acceptable among populations at greatest risk of HIV infection.
